# Chitosan, Chitosan/IgG-Loaded, and N-Trimethyl Chitosan Chloride Nanoparticles as Potential Adjuvant and Carrier-Delivery Systems

**DOI:** 10.3390/molecules28104107

**Published:** 2023-05-15

**Authors:** Aldo Y. Tenorio-Barajas, María de la L. Olvera, Gabriel Romero-Paredes, Victor Altuzar, Efraín Garrido-Guerrero, Claudia Mendoza-Barrera

**Affiliations:** 1 Doctorado en Nanociencias y Nanotecnología, Centro de Investigación y Estudios Avanzados del IPN, Ciudad de México 07360, Mexico; 2 Facultad de Ciencias Físico Matemáticas, Benemérita Universidad Autónoma de Puebla, Puebla 72570, Mexico; 3 Sección de Estado Sólido, Departamento de Ingeniería Eléctrica, Centro de Investigación y de Estudios Avanzados del IPN, Ciudad de México 07360, Mexico; 4 Departamento de Genética y Biología Celular, Centro de Investigación y Estudios Avanzados del IPN, Ciudad de México 07360, Mexico

**Keywords:** chitosan, loaded nanoparticles, microfluidics, *in vitro* culture

## Abstract

This work proposes a feasible, reproducible, and low-cost modified method to manufacture chitosan, chitosan/IgG-protein-loaded, and trimethylated chitosan nanoparticles, using microfluidics combined with the microemulsion technique, which differs from the traditional batch process of chitosan-based nanoparticles. The synthesis process consists of generating microreactors of chitosan-based polymer in a poly-dimethylsiloxane ψ-shaped microfluidic device and then crosslinking with sodium tripolyphosphate outside the cell. Transmission electron microscopy demonstrates an improvement in size control and distribution of the solid-shape chitosan nanoparticles (~80 nm) compared to the batch synthesis. Regarding chitosan/IgG-protein-loaded nanoparticles, these presented a core-shell morphology having a diameter of close to 15 nm. Raman and X-ray photoelectron spectroscopies confirmed the ionic crosslinking between the amino groups of chitosan and the phosphate groups of sodium tripolyphosphate in the fabricated samples and the total encapsulation of IgG protein during the fabrication of chitosan/IgG-loaded nanoparticles. Then, an ionic crosslinking and nucleation-diffusion process of chitosan-sodium tripolyphosphate was carried out during the nanoparticle formation, with and without IgG protein loading. The use of N-trimethyl chloride chitosan nanoparticles *in vitro* on human-keratinocyte-derived cell line HaCaT did not show side effects independently of its concentration from 1 to 10 μg/mL. Therefore, the proposed materials could be used as potential carrier-delivery systems.

## 1. Introduction

Chitosan (CS) is a derivative of chitin, which is the second most common biopolymer in nature after cellulose. The principal raw sources for chitosan preparation are crustacean shells, such as those of shrimp, crabs, lobster, crayfish, and oysters, due to their 30–75% weight content of chitin, although other species, such as insects and fungi, contain this biopolymer [1,2,3,4]. Since shells represent 40–50% of the weight of the total mass of the crustacean, human consumption could lead to significant pollution in coastal areas; therefore, uses and reuses of chitin for industrial applications are fundamental [5,6].

Chitosan is a linear polysaccharide composed of *N*-acetyl-d-glucosamine and d-glucosamine with a strong positive charge. Moreover, it is a biocompatible, biodegradable, and non-toxic biopolymer, and an excellent candidate for a wide range of biological and biomedical applications, including tissue engineering, wound healing, and gene or drug delivery [6]. Chitosan nanoparticle (CS-NP) production is commonly achieved in batch synthesis by crosslinking chitosan dissolved in an acidic solution with pH values from 3 to 6 and then crosslinking with sodium tripolyphosphate (TPP), as first reported by [7]. The spontaneous formation of nanoparticles is due to electrostatic forces between amino groups from CS and the TPP [7,8]. Other fabrication processes, such as polyelectrolyte complex, microemulsion, incorporation and incubation, solvent evaporation, coprecipitation, and complex-coacervation methods, have been used to fabricate chitosan and chitosan-based nanoparticles. Significantly, the microemulsion method allows the fabrication of chitosan and chitosan-alginate core-shell nanoparticles when the nanoparticles are formed in the aqueous core of the reverse micelle using a cosurfactant and a crosslinking agent [9]. Some properties of chitosan, such as its positive charge and solubility in aqueous solutions, provide affinity to negatively charged materials such as proteins, lipids, and DNA. Other subproducts of chitosan molecules have been synthesized, such as water-soluble CS obtained through methylation [10] and hydrophobic chitosan to encapsulate hydrophobic drugs and improve immune response [11].

Fabrication using microfluidics systems offers new ways to synthesize nanoparticles in a narrowly controlled manner with many advantages over the traditional batch synthesis method [12], including low reagent consumption, high uniformity, and narrow size distribution of several types of particles. The biological area has the most significant applicability potential of microfluidics, such as in encapsulating possible organic compounds or active ingredients. Nevertheless, the lack of microfluidic fabrication is due to the low production rate of the nanostructure of interest. 

In previous work, we proposed using chitosan nanoparticles, generated by the batch synthesis method, as an adjuvant since we demonstrated that CS nanoparticles present better adjuvant properties than alumina in mice, without its disadvantages [13]. However, one challenge exists for further adjuvant or nanocarrier applications because the batch process creates heterogeneous morphologies and broad-size distribution of nanoparticles. Therefore, in this paper, we provide a feasible, reproducible, and low-cost modified method using microfluidics combined with the microemulsion technique to synthesize chitosan and chitosan/IgG-protein-loaded nanoparticles. First, we describe the manufacture of the microfluidic cell in poly-dimethylsiloxane (PDMS) using a standard photolithography microfabrication process. Then, the synthesis and morphological, compositional, and surface characterization of chitosan nanoparticles (CS-NPs) and chitosan human IgG loaded nanoparticles (CS/IgG-NPs) are described. The fabricated nanoparticles and nanocarriers showed a narrower size distribution when using the proposed synthesis procedure than those generated using the traditional batch method. Finally, methylated chitosan was used to produce N-trimethyl chitosan chloride IgG protein loaded nanoparticles (TMCS/IgG-NPs), which were evaluated in the human keratinocyte-derived cell line HaCaT *in vitro*.

## 2. Results and Discussion

### 2.1. Microfluidic Device Fabrication

**Figure 1:**
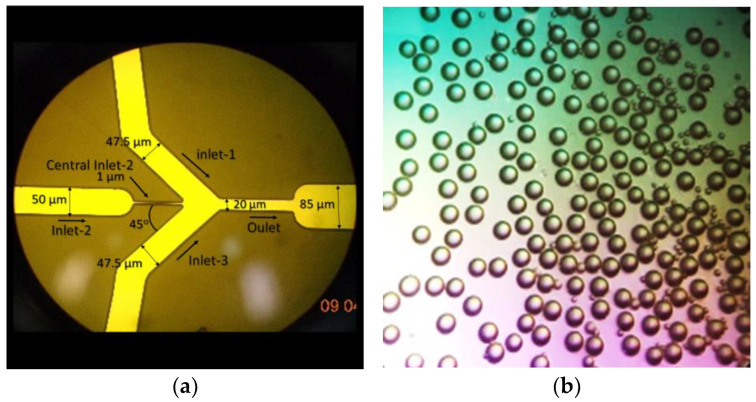
(**a**) Microchannels fabricated on glass under an optical microscope, determined using profilometry; (**b**) optical observation of CS-NP drop microreactors.

Several methods exist for drop formation or nanoparticle fabrication through microfluidics; however, the photolithograph produces one of the best results. Nevertheless, using capillaries in co-flow for particle production is a high-control method [14], which could be an inexpensive method for micro-drop production, avoiding clean rooms and lengthy processes requiring toxic and dangerous chemicals. We used this technology to fabricate ψ-shape microfluidic PDMS microchannels using a master-glass microchannel pattern (Figure 1a) which allowed us to easily replicate the channels to manufacture new microfluidic cells in PDMS. Then, we combined it with the batch technique to produce CS-based nanoparticles. 

Profilometry results showed that the inlet-2 channel has a width of 50 μm and a depth of 9 μm, whereas the outlet channel has a diameter of 20 μm and a depth of 5 μm. Moreover, central inlet-2, inlet-1, and inlet-3 channels, which converge at the hydrodynamic flow, have widths of 1, 50, and 50 μm, respectively, and a diameter of 5. The angles of the fabrication of inlet-1 and intel-3 channels were 45° concerning the central inlet-2 channel. At its side, the inlet-1 and inlet-3 channels assemble at the cross-section and focus the hydrodynamic flow in the primary intel-2 channel to produce drops.

### 2.2. Synthesis of CS and CS/IgG-Loaded Nanoparticles

**Figure 2:**
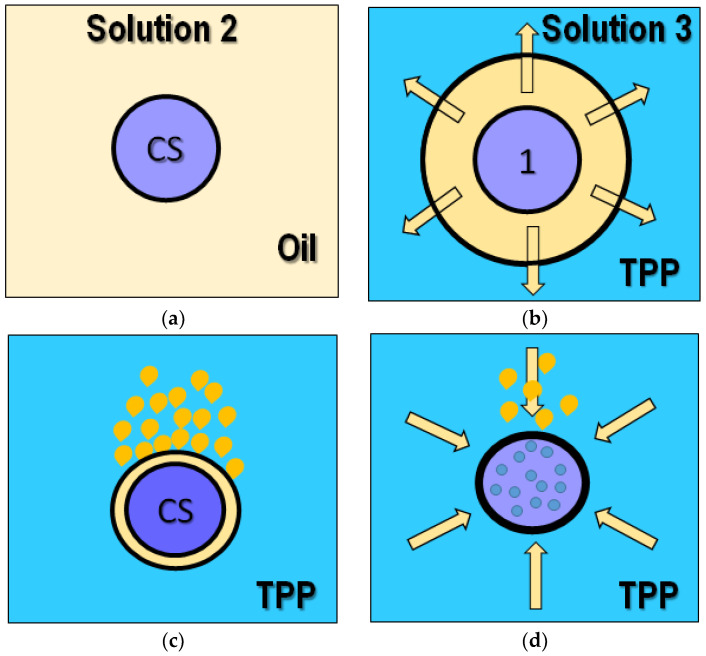
The proposed mechanism for nanoparticle nucleation and formation inside micro-drop reactors of CS under soft mechanical stirring. (**a**) Micro-drops of CS in oil generated inside of the microfluidic device; (**b**) micro-drops of CS in oil dropped into a TPP solution out of the microfluidic device; (**c**) oil capping starts to thin; (**d**) crosslinking of CS material inside the CS drop by diffusion to obtain CS-NPs.

CS and IgG-loaded nanoparticles were fabricated using the fabricated ψ-shape microfluidic PDMS/glass device; Figure 1. We observed highly uniform dispersed drops of chitosan formation in the oil carrier fluid during fabrication at the outlet pipe; Figure 1b. The proposed CS nanoparticle formation mechanism is schematized in Figure 2.

Initially, in Figure 2a, the microfluidic device produces micro-drops of CS (solution 1) in oil (solution 2), which are transported to the hosepipe out of the microfluidic device and deposited into the TPP solution (solution 3); Figure 2b. The difference in densities between solutions produces oil capping around the CS drops. In contrast, the TPP from surrounding media starts to migrate by diffusion and gradient concentration difference between the CS drops and the surrounding media as soon as the oil layer becomes thinner; Figure 2c. Using the crosslinker outside the microfluidic device avoids the clogging of microchannels, whereas the CS micro-drops act like a microreactor for CS nanoparticle nucleation and formation; Figure 2d. 

Five CS solutions (0.01, 0.1, 0.2, 0.5, and 1% *w*/*v*) in acetic acid (1%, pH 3, [7]) were used as the hydrophilic dispersed phase to produce CS drops with the PDMS microfluidic chip, whereas mineral oil was the hydrophobic carrier fluid. The abovementioned CS and oil carrier fluid were injected into the microfluidic chip to determine, under microscope observation, the CS concentration solution and pressure conditions for the CS and oil, leading to a uniform CS drop formation (Figure 1b). 

**Figure 3:**
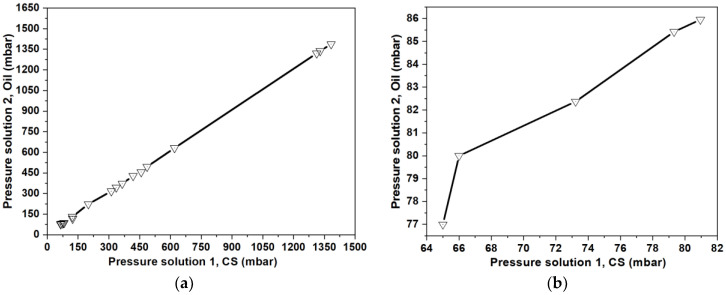
(**a**) Pressure conditions (hydrophilic CS and hydrophobic solutions) for the CS drop formation inside the ψ-shape PDMS microfluidic chip; (**b**) detail of the non-linear region of the pressures of (**a**), in the interval 65 to 81 mbar.

Figure 3 shows the CS and oil pressure sets for the CS drop formation inside the ψ-shape PDMS microfluidic chip with a CS solution concentration of 0.5 % *w*/*v*. Higher CS concentration values lead to an occlusion of microchannels in the chip, whereas lower concentrations generate CS drops of several sizes. Above 74 mbar, a linear relationship between CS and oil solution was found (Figure 3b), independent of the CS concentration used. In addition, higher pressures imply lower CS-drop diameter; however, future studies are needed to determine the type of relationship, size, and dispersion of the produced particles. We selected pressures in the lineal region presenting higher drop uniformity under the microscope. In the case of CS-NP, we used 270 and 282 mbar for the CS and oil flows, respectively, while 290 and 298 mbar were used during the fabrication of CS/IgG-NP.

### 2.3. Characterization of CS and CS/IgG-Loaded Nanoparticles

**Figure 4:**
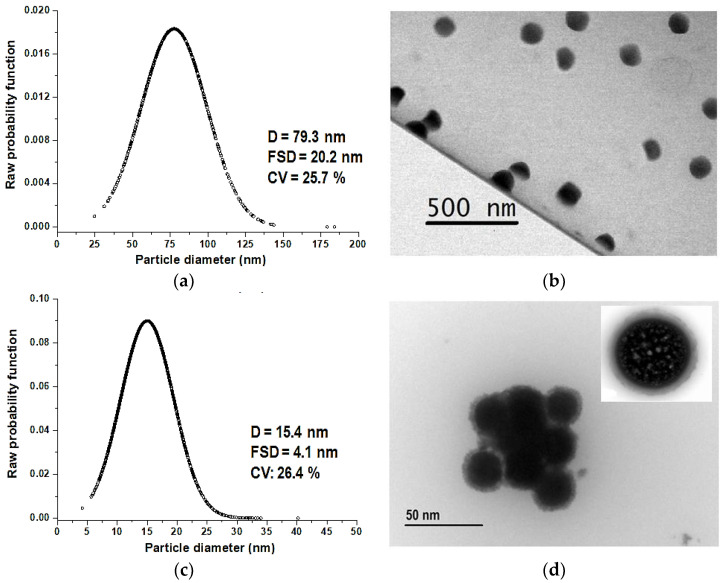
Average diameter D, first standard deviation FSD, and coefficient of deviation CV of (**a**) CS-NP; (**b**) CS/IgG-NP. TEM micrographs of (**c**) CS-NP; (**d**) CS/Ig-NP.

Figure 4b shows the TEM micrographs of CS-NPs, whereas Figure 4d corresponds to CS nanoparticles loaded with human IgG, CS/IgG-NPs. We observed morphological differences between CS nanoparticles and those samples loaded with IgG. CS-NPs have a solid and uniform appearance, whereas those laden with protein present a crown with a central nucleus.

We determined that nanoparticles loaded with IgG protein were five times smaller than the CS nanoparticles. IgG protein within CS micro-drops probably acts as a nucleation center for nanoparticle formation, inducing the production of smaller particles of CS compared with nanoparticles without protein.

The average diameter D, first standard deviation FSD, and the coefficient of deviation CV were calculated from the TEM micrographs; Figure 4a,c and Table 1.

The variation coefficient in both cases was close to 25% in both fabricated samples.

**Figure 5:**
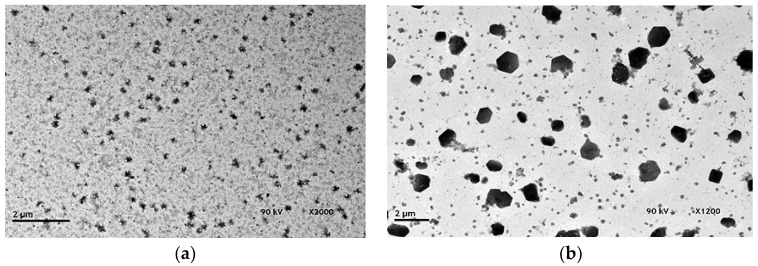
TEM micrographs of (**a**) CS-NP (v = 15 mL/h); (**b**) CS-NP (v = 70 mL/h) fabricated by the batch route.

For comparison between fabrication processes, Figure 5 presents the TEM results of CS samples fabricated with the traditional batch process [8,9] when the CS solution was added under constant rates of 15 and 70 mL/h.

**Figure 6:**
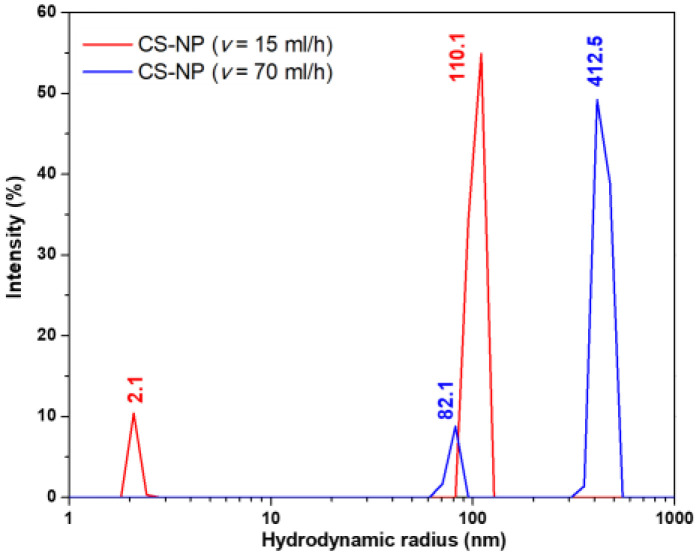
DLS of CS-NP (v = 15 mL/h) and CS-NP (v = 70 mL/h) fabricated by the batch route.

The average hydrodynamic radius R_hyd_ was determined using Dispersive Light Scattering (DLS; Figure 6). We found two different groups of particles with random morphologies in each sample. Sample CS-NP (v = 15 mL/h) presented 2.1 and 110.1 nm particles, whereas CS-NP (v = 70 mL/h) showed a bigger R_hyd_ of 82.1 and 412.5 nm. Then, microfluidic fabrication improved the morphology, uniformity, and particle size over the batch process, besides the possibility of encapsulating and transporting cargo. Moreover, the combined use of microfluidics assisted by the emulsification process allows higher amounts of fabricated material than microfluidic synthesis alone. 

**Figure 7:**
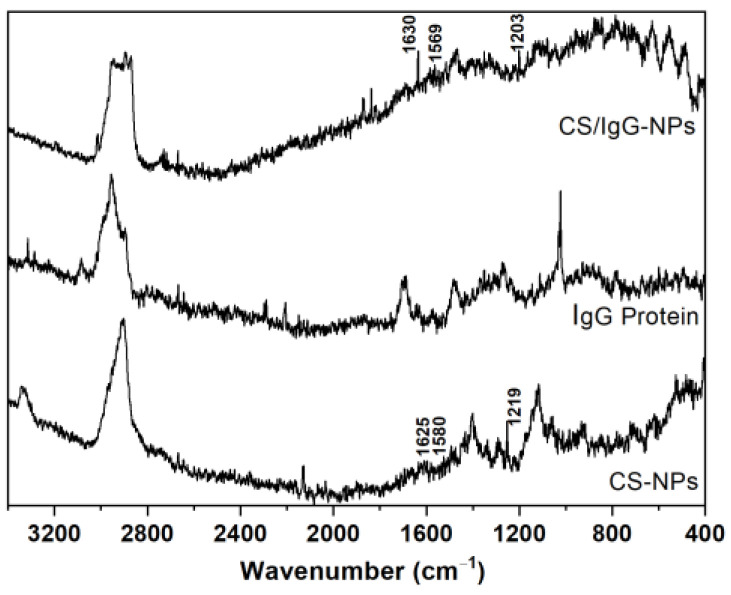
Raman spectra of CS-NP and CS/IgG-NP. IgG protein was used as a control.

Figure 7 presents the Raman spectra of IgG native protein, CS-NP, and CS/IgG-NP samples fabricated with microfluidics assisted by emulsification. The strongest characteristic vibrational bands from human IgG are due to the vibrations of the aromatic side chains of phenylalanine (Phe), tryptophan (Trp), and tyrosine (Tyr), as well as to the amide III and I’ vibrations of the β-sheet structure. The bands for native protein were found at 2860 and 2940 cm^−1^ (νC-H bonds), 1705 cm^−1^ (amide I’), 1483 (CH_2_ bending mode), 1273 (amide III of β-sheet structure), 1023 (C-N), 1014 (Phe), and 995 (C-C stretch) [15,16]. The shift was attributed to protein denaturation, which usually presents weaker and broader amide III and amide I’ bands, indicating decreased β-sheet structure and an untidier conformation [16]. In addition, the principal vibrational bands of CS-based samples were found at 2885 (CH_2_ stretching mode), 1654 (C=O stretching band), 1591 (in-plane bending NH_2_ vibration), 1430 (bending vibration of O-H), 1370 (bending vibration of C-H), 1030 and 1080 (skeletal vibrations involving O-C stretching), 936 (C-N stretching band), and 896 (stretching pyranoid ring and CH_2_ bending mode) cm^−1^ [17,18]. Furthermore, it has been reported that TPP presents at 1210 cm^−1^ the stretching vibration of P=O, and at 1130 and 1090 are the symmetric and anti-symmetric stretching vibrations in the O−P=O and PO_3_ groups, respectively, whereas 888 cm^−1^ can be assigned to the asymmetric stretching vibration of the P−O−P bridge [18].

We observed the peaks associated with P=O of TPP in CS-NPs and CS/IgG-NPs at 1219 and 1203, respectively, due to the crosslinking between CS and TPP. The bands for N-H of amine I and amide II carbonyl vibration stretches were shifted to 1580 and 1625 for CS-NPs, whereas CS/IgG-NP samples were at 1569 and 1630 cm^−1^. Then, the interaction between amino and phosphate groups of CS and TPP was carried out [19]. The peak from IgG protein at 1705 cm^−1^ is also present in the CS/IgG spectrum, confirming its presence in the sample. 

**Figure 8:**
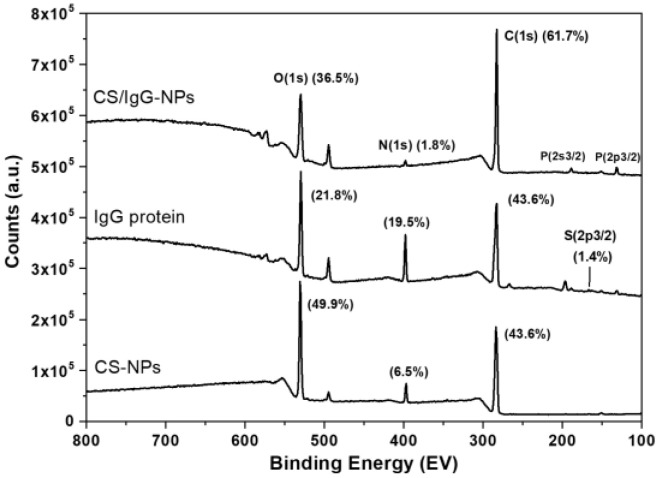
XPS survey of CS-NP and CS/IgG-NP samples. IgG protein was included as a control.

Due to the similarity between functional groups from CS and IgG, we used XPS to determine the difference in elements between CS and IgG. Since IgG is a 150 kDa Y-type protein with a length of 10 nm, XPS is helpful to establish if CS has encapsulated inside the IgG protein [20]. Figure 8 shows the XPS surveys of CS-NP and CS/IgG-NP samples. 

**Figure 9:**
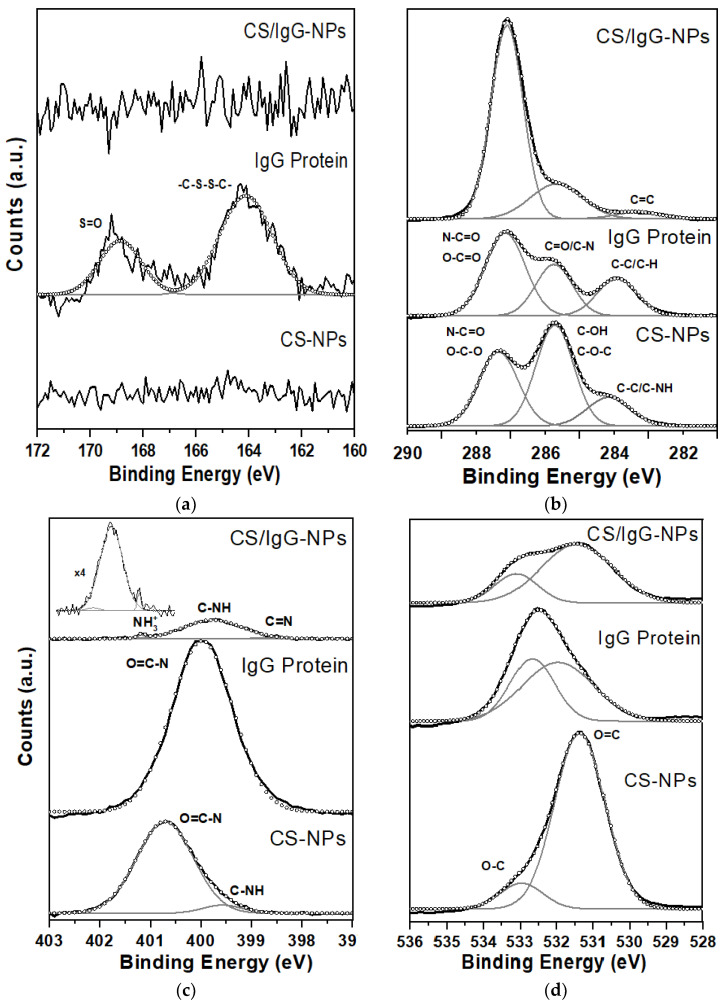
XPS detailed spectra of CS-NP and CS/IgG-NP. (**a**) S(2p3/2); (**b**) C(1s); (**c**) N(1s); (**d**) O(1s) peaks. IgG protein was used as a control.

IgG protein was also used as a control here. We observed the usual O(1s) and C(1s) peaks at 532 and 285 eV, respectively. As expected, N(1s) at 399 eV is also present in all samples due to CS and IgG. Moreover, a small S(2p3/2) double peak around 164 eV in IgG protein is detected, corresponding to their disulfide bridges [21]. The CS/IgG-NP sample showed the presence of P(2p3/2) and P(2s3/2) at 132 and 190 eV, respectively, assigned to the PO of TPP during the crosslinking with CS. Nevertheless, the CS-NP sample showed a small peak at 132 eV, indicating a poor crosslinking of TPP with CS [22]. The atomic concentration (%) for S(2p3/2), C(1s), N(1s), and O(1s) for CS-NP and CS/IgG-NP samples was determined, including IgG protein as a control. Neither CS-NP nor CS/IgG-NP showed the presence of sulfur, whereas the IgG protein showed 1.4%. The C(1s) concentration increased from 43.6 to 61.7%, while the N(1s) decreased from 6.5 to 1.8% in the CS-NP and CS/IgG-NP samples. When the protein was added, the C(1s) increment could be explained by the sample CS/IgG-NP acetylation. The opposite behavior has been reported during the fabrication of chitosan nanoparticles when the nitrogen content is increased [23,24]. Moreover, this confirms the exposure of nitrogen on the surface of the CS/IgG-NP coming from CS. High-resolution XPS spectra and deconvolution of S(2p3/2), C(1s), and N(1s) peaks from CS-NP, IgG protein, and CS/IgG-NP samples are shown in Figure 9 and Table 2.

We used the S(2p3/2) orbital as a unique signature of the elemental states of the samples to find out if the protein was inside or outside of the CS particles [20,25]. Only the IgG protein sample showed the presence of sulfur. This means total protein encapsulation during CS/IgG-NP fabrication, as suggested by TEM results. The S(2p3/2) spectrum was resolved into two peaks at 168.9 and 164.1 eV. The first peak was assigned to the S=O bond, whereas the second corresponds to the disulfide bridge (-C-S-S-C-) bonding state from the 0.19% of Cysteine and Methionine amino acids of the structure of the protein; Figure 9a [26,27]. The C(1s) peak of the analyzed samples was resolved into three main peaks; Figure 9b. The IgG sample presented at 284.9 eV the aliphatic hydrocarbon C-H bond, whereas at 286.7 eV, the carbon was bound to oxygen (C-O) or nitrogen (C-N), which are characteristics of the carbon of IgG protein (NH-CHR-CO) or their residues. The last peak at 288.2 eV is the fingerprint of the proteins, which could be assigned to the amide carbon (N-C=O) [23,28]. In addition, the CS-NP sample presented as the first peak of the aliphatic C-C carbon, C-N, and any adventitious carbon at 285.1 eV; alcohol (C-OH) and ether (C-O-C) bonds at 286.7 eV; and O-C-O and amide carbonyl (N-C=O) groups at 288.3 eV [29]. In particular, the C=O bond can be attributed to acetyl groups from the CS backbone and the TPP crosslinker [30]. We found that C(1s) of the CS/IgG-NP sample showed the same groups present in CS/NPs, with a slight position shift at C-C/C-H groups and a ratio peak change. Since we did not find any C-S group, -C-S-S-C-, or S=O in the C(1s) and S(2p3/2) spectra in this sample, we determined that the IgG protein was encapsulated inside the nanoparticles. 

Figure 9c shows the characteristic N(1s) peak of proteins at 400 eV [18,31] of CS-NP and CS/IgG-NP samples, resolved into two and three peaks, respectively. CS-NPs showed the O=C-N amide carbonyl group at 400.7 eV, whereas at 399.6 eV, the C-NH group was assigned. For their part, CS/IgG-NPs presented positively charged nitrogen NH^3+^ at 401 eV, the C-NH group at 399.7 eV, and the C=N bond at 398.8 eV [23,32]. All O(1s) spectra were deconvoluted in two peaks because the ether and alcohol groups have similar binding energies [23,26,33]; Figure 9d. IgG protein showed C=O and C-O groups at 531.9 and 532.7 eV; CS-NP at 531.5 and 532.9 eV; whereas CS/IgG-NPs were at 531.4 and 533.2 eV, respectively. The increase in N-C=O content at 288 eV in C(1s) and the new C=N peak of N(1s) in 398.8 eV spectra when the IgG is loaded into the CS nanoparticle confirms the strong wrapping of carbon and nitrogen bonds from CS polymer and IgG protein. This could explain the narrow normal distribution and the smallest size of CS/IgG-NPs produced against the CS-NPs. 

### 2.4. N-Trimethyl Chitosan Chloride Nanoparticles Toxicity in Human Keratinocyte (HaCaT) Cells

**Figure 10:**
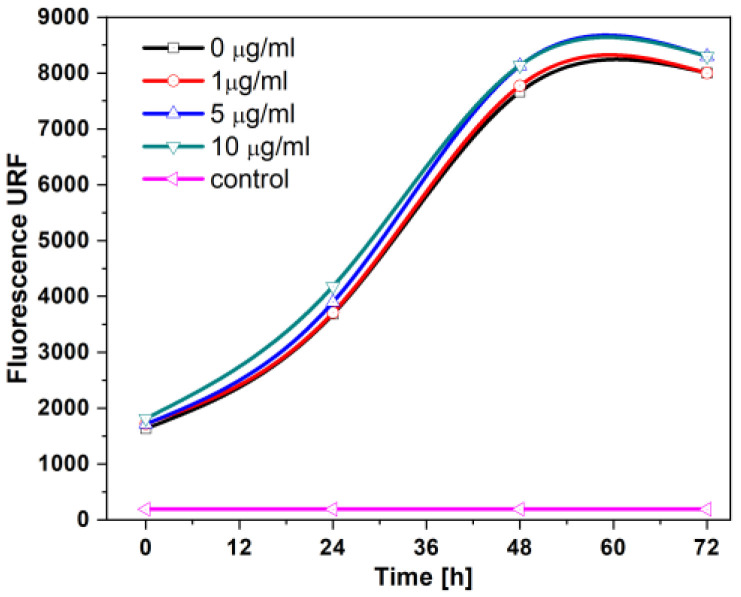
Fluorescence level 570/600 nm (URF) was detected in the assays of TMCS/IgG-NP treatment on the proliferation rate of HaCaT cells. Alamar Blue assay was performed on HaCaT cell cultures after treatment with 0, 1,5, and 10 μg/mL of TMCS-NP for the indicated time.

Biocompatibility is one of the most critical characteristics in nanoparticles with adjuvant potential since the toxicity of the materials can induce a modification of cell membranes or a change in cellular metabolism. The cell proliferation rate was evaluated using HaCaT cells exposed at 0, 1, 5, and 10 μg/mL of TMCS/IgG-NP for 72 h; Figure 10. We observed the non-toxic effect of the cells with nanoparticle treatment since the proliferation rate was very similar regardless of the different concentrations used, compared to the one found in control cells without nanoparticle treatment. Moreover, higher levels of TMCS/IgG-NP (25, 50, and 75 μg/mL) were tested, and neither caused a toxic effect in the HaCaT cells (results not shown here). Although the number of cells seeded in each plate allowed a culture for 72 h, a slight but consistent increase in the reduction value of Alamar Blue was observed with 5 and 10 μg/mL treatments at 24 and 48 h, indicating that TMCS-NPs not only do not only affect cell viability but also slightly induce an increase in oxidative metabolism in this cell line. This could be due to possible endocytosis of the NPs, since their size and cationic nature would allow them to adhere to the negative cell surface, facilitating their uptake and increasing mitochondrial metabolism. More experiments are needed to establish the molecular mechanism of this behavior. In accordance with our findings, Amidi et al., 2006 [34] reported that TMCS-NPs had no cytotoxic effects on a cell line derived from lung adenocarcinoma (Calu-3 cells), even at the highest concentration evaluated in that study (40 mg/mL). Although the non-toxicity of TMCS/IgG-NP was expected [35,36], this was an encouraging result since it provides the possibility of further studies in other cellular models, immunological assays, or even *in vivo* systems.

In summary, the microfluidics of the continuous flow and the microemulsification methods, focusing on nanoparticle production for biomedicine applications, have been reported previously [9,37,38,39]. Here, we successfully fabricate, from a reusable biopolymer such as chitosan, a derivative of chitin, CS, CS-IgG protein-loaded, and TMCS-IgG protein-loaded nanoparticles using the microfluidics assisted by an emulsification method. The combination of both processes inherits their benefits to reach control and high-rate production of nanoparticles. The mechanism proposed here takes advantage of microfluidic control of water/oil drop production to precisely scale up the distribution and reproducibility during the fabrication of CS-based nanoparticles without downscaling the photolithographic process to the nanoscale, which tends to increase the issues during fabrication and instrumentation. We improve the dispersion of the CS-NPs fabricated with the microfluidics assisted by the emulsification method in contrast with those fabricated with the traditional batch synthesis process. In the CS/IgG protein-loaded nanoparticles, we demonstrated that IgG protein works as a nucleation center during CS/IgG-NP formation since we found the total encapsulation of IgG and a core-shell morphology close to 15 nm. In addition, CS-NPs presented a solid shape having a diameter of ~80 nm and the ionic crosslinking between the amino groups of chitosan and the phosphate groups of sodium tripolyphosphate. Moreover, N-trimethyl chloride chitosan nanoparticles, fabricated with the proposed route of *in vitro* cultures on the human-keratinocyte-derived cell line, HaCaT, did not show side effects independently of the 1~10 μg/mL concentration used. 

## 3. Materials and Methods

### 3.1. Fabrication of the Microfluidic Device

**Figure 11:**
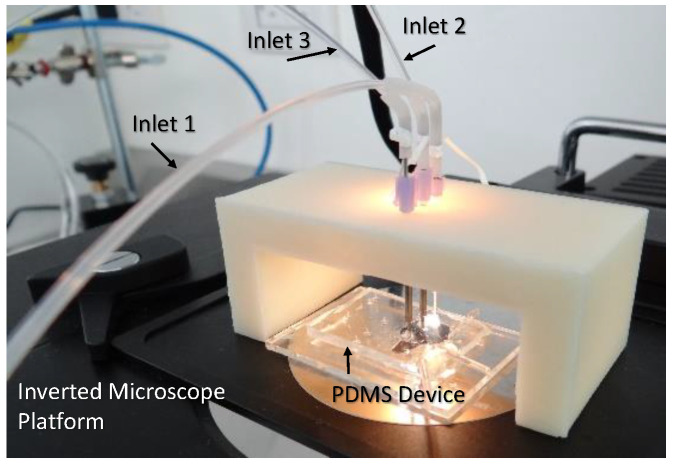
PDMS device connected to a homemade injection system.

A Ψ-shape microfluidic design was fabricated in PDMS/glass, with three flow inlet microchannels that converge into an outlet channel. Contact profilometry (Dektak 150, Veeco Instruments, Plainview, NY, USA) was used to verify the width and depth of the flow in the microchannel of the three inlets and the outlet microchannel. A flat glass master with multiple microchannel patterns was previously fabricated using a standard photolithographic microfabrication process. Briefly, a highly flat glass (Schott B270) was cleaned using the RCA process, and then it was covered with a 100 nm layer of Chromium (Cr) by sputtering (V1, Intercovamex). Then a layer of micro-positive resin SC1827 (Rohm and Hass Electronic Materials LLC, Marlborough, MA, USA) of 1.5 μm thickness was spin-coated (KW-4E, Setcas L.L.C., San Diego, CA, USA) at 4000 rpm for 20 s and baked at 90 °C for 20 min (Blue M Electric oven). A laser writer (Heidelberg μ-PG-101) was used to generate the pattern. Then, the design over the resin was revealed (C.A.S. 7732, Burlington, MA-18-5, JT Baker, Avantor Inc., Radnor, PA, USA) and baked again (20 min, 120 °C). Then, the Cr was exposed to chromium etchant (CAS 651826, Sigma Aldrich, Burlington, MA, USA). Finally, the back of the glass master was covered with resin and baked at 120 °C for 20 min, and etched for 5, 10, 15, 20, 25, 30, and 35 min with H_2_O, HF Buffer (NH_4_F, 10:1), and HCl in a proportion of 17:1:2. The master pattern was copied with PDMS Sylgard^®^ 184 in the conventional procedure: a mixture of PDMS and the curing agent (10:1) was mixed, degassed, and poured over the master mold and immediately baked at 70 °C for 2 h. The resulting PDMS device was removed from the glass master, and the ports were punched to connect the hosepipe. A flat glass (Schott B270) was used to cover up the PDMS device. Both sides of the device were exposed to O_2_ plasma for 30 s in a Reactive Ion Etching (RIE) camera (PE100, Plasma Etch Inc., Carson City, NV, USA) and sealed to produce the final microfluidic device (Figure 11). A homemade 4-injection syringe system [40] was used to inject the fluids into the microchannel cell.

### 3.2. Synthesis of Chitosan and Chitosan Human IgG Loaded Nanoparticles by Microfluidics Assisted by Emulsification

**Figure 12:**
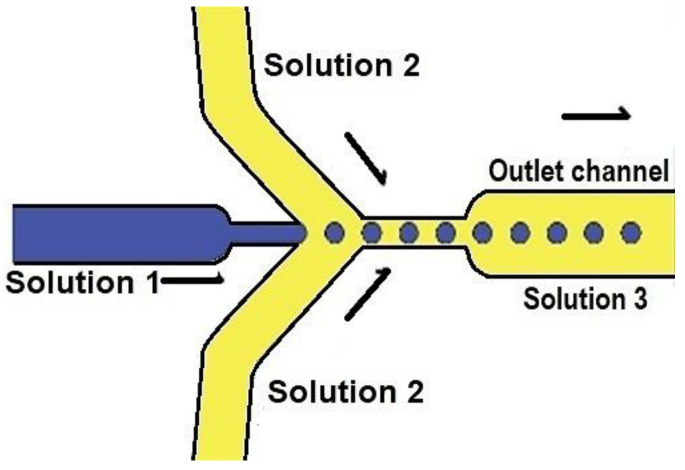
Configuration for micro-drop chitosan production inside the ψ-shaped microfluidic PDMS/glass device.

CS of low molecular weight (50–190 kDa, CAS 9012-76-4, Sigma Aldrich, Burlington, MA, USA), with a deacetylation degree (DD) of 85% at 0.01, 0.1, 0.2, 0.5, and 1% *w*/*v* concentrations, was dissolved in a solution of acetic acid (1%, pH 3) according to [7]. All solutions were stirred at room temperature for 12 h. Then, the CS solutions (Solution 1) were filtered (0.20 μm mesh, NPMillipore) and used as the hydrophilic dispersed phase to produce CS particles using the fabricated microfluidic chip. In contrast, mineral oil was the hydrophobic carrier fluid. The CS and oil carrier fluid were injected into the ψ-microfluidic chip (Figure 11 and Figure 12) to determine, under microscope observation, the CS concentration solution and pressure conditions for the CS and oil, leading to uniform CS drop formation (Figure 1b). A second solution at 2% *v*/*v* (Solution 2) of mineral oil (CAS M8410) with Span 80 was used as a hydrophobic carrier fluid. Solution 2 was dripped from the microfluidic device into a beaker glass with TPP in DI water (10% *w*/*v*) to crosslink it. The crosslinked solution was named Solution 3 and was centrifuged at 5000 rpm (1960× *g*) for 5 min to separate oil hydrophobic and aqueous hydrophilic phases (Figure 12). The CS centrifuged particles were resuspended with DI water, Solution 4, and stored until their characterization.

In the case of CS/IgG nanoparticle production (CS/IgG-NP), 1 mg/mL of human IgG protein (Cat 401114, Lot D00030076, Calbiochem Merck, Darmstadt, Germany) was added to the CS solution (Solution 1), and a PDMS Ψ-shape copy microfluidic device was used, following the previously described process. To compare the proposed synthesis process, we also fabricated CS particles using the traditional protocol reported in [8,9] with slight modifications. Briefly, a TPP solution was placed in a beaker glass under vigorous mechanical stirring, whereas a CS solution was added by dropping it with a syringe pump (KDS100) at 15 and 70 mL/h rates with a 1:5 *v*/*v* ratio. The resulting solution was centrifuged at 2570× *g* (5725 rpm) for 1 h, the supernatant was removed, and the remaining solution was freeze dried (Labconco) at −50 °C for 24 h.

### 3.3. Characterization of CS and CS/IgG Nanoparticles

Transmission electron microscopy (TEM, JEM-ARM200F) was used to visualize the morphological characteristics of the fabricated samples and to determine their average diameter. They were negatively stained with uranyl acetate (2% *v*/*v*) [40] before observation. The samples were dispersed and dropped directly on a formvar/carbon grid supported by a Cu mesh (TedPella Inc., Redding, CA, USA). The average diameter, standard deviation, and variation coefficient in CS-NP and CS/IgG-NP samples were calculated using 1000 particles in 4 TEM micrographs (×80,000) of each sample. Raman (Jobin-Y von T64000) spectroscopy was carried out to determine the vibrational bands of the samples, as well as for IgG protein as a control. It was performed in absorption mode from 400 to 3500 cm^−1^ with the sample embedded in a KBr tablet. To determine the possible encapsulation of IgG protein inside the NP during the microfluidic synthesis process, we analyzed CS-NP, CS/IgG-NP, and IgG protein (1 mg/mL) samples by XPS (K-Alpha Thermo Fisher Scientific Inc., Waltham, MA, USA) in survey and detail modes. We used the C-H peak at 285 eV (277–294 eV, analyzer pass energy = 50 eV) as a reference [20,31]. The survey spectra were used to calculate the atomic compositions from atomic orbitals C 1 s, O 1 s, N 1 s, and S 2p3/2. The CS samples fabricated using the batch process were characterized by TEM and Dynamic Light Scattering (DLS). DLS measurements were performed using a Zetasizer Nano S instrument (Malvern Instruments) at room temperature (25 ± 0.1 °C). 

### 3.4. Toxicity of N-Trimethyl Chitosan Chloride Nanoparticles Synthesized by Microfluidics Assisted by Emulsification TMCS-NP Toxicity in Human Keratinocyte (HaCaT) Cells 

To test CS-based nanoparticles of interest in a biological model, we produced N-trimethyl chitosan chloride (TMCS) according to [11], with a yield of 56% (results not shown here). TMCS and TMCS/IgG nanoparticles were fabricated following the aforementioned microfluidics assisted by the emulsification method. The obtained TMCS/IgG-NPs were lyophilized and resuspended in deionized water at 1, 5, and 10 μg/mL. The high solubility of TMCS in water (pH 7.0) suggested potential compatibility of the generated TMCS/IgG-NPs with standard conditions used in cell culture and perhaps with cell growth. We used the spontaneously immortalized human skin keratinocyte cell line HaCaT [19] to test them. The cell line was grown in culture dishes in Dulbecco’s modified Eagle’s medium (DMEM, Invitrogen, Carlsbad, CA, USA) supplemented with 10% fetal bovine serum (FBS), L-glutamine (2 mM), sodium pyruvate (1 mM), penicillin (50 U/mL), and streptomycin (50 µg/mL), in a humidified atmosphere with 5% CO_2_ at 37 °C and maintained in an exponential growth phase. Exposure of HaCaT cells to TMCS-NPs was performed, seeding 3 × 10^4^ cells/well (on a 24-well plate) and, 24 h later, adding 1, 5, or 10 µg/mL of TMCS-NP to the cultures. The Alamar Blue reduction method was used to evaluate the effects on cell proliferation of nanoparticle exposition after 4, 24, 48, and 72 h. Cellular monolayers were washed with phosphate buffered saline (PBS 1×), and a mix of Opti-MEM Reduced Serum Media (Invitrogen) and Alamar Blue reagent (CAS 62758-13-8, Bio-Rad) (9:1) was added to each cell well and incubated for 4 h. Then, a 150 µL aliquot from the medium was taken from each well and transferred to a 96 non-translucid well plate. To determine the number and metabolic activity of living cells in the well, their fluorescence, at 570 nm excitation and 600 nm emission wavelengths, was measured in fluorometer equipment (Fluoroskan Ascent FL, Thermo Fisher Scientific Inc., Waltham, MA, USA). 

## 4. Conclusions

In this work, using the standard photolithographic process in PDMS/glass, a Ψ-shape microfluidic cell was manufactured, including three flow inlet microchannels that converge into an outlet channel. The cell was used to fabricate chitosan, CS-IgG protein-loaded, and TMCS-IgG protein-loaded nanoparticles using microfluidics assisted by an emulsification method. It was evidenced from the characterization results that the continuous flow regime combined with the microemulsification process during nanoparticle production and protein encapsulation improves the size control and distribution of the chitosan-based nanoparticles compared to the traditional batch synthesis method, while allowing a similar amount of nanoparticle production. The droplet regime inside the microfluidic device produces uniform water in oil microreactors, improving the size dispersion of nanoparticles during the crosslinking outside the cell. Chitosan nanoparticles showed a higher solid-shape diameter than chitosan/IgG-loaded nanoparticles, which presented a core-shell morphology with the total encapsulation of IgG protein. In both cases, the crosslinking between the amino groups of chitosan and the phosphate groups of sodium tripolyphosphate was carried out. The *in vitro* studies of water-soluble CS nanoparticles indicate no cytotoxic effects on the human-keratinocyte-derived cell line HaCaT independently of its concentration. The slight increase in the oxidative metabolism induced by TMCS-NP treatments suggests an endocytic process. The validation of both observations in other cellular models would allow visualizing potential applications of these nanoparticles, both in transporting molecules into the cell, as well as in immunological assays and *in vivo* models.

## Figures and Tables

**Table 1:** Average diameter D, first standard deviation FSD, and coefficient of deviation CV of CS-NP and CS/IgG-NP.

Sample	Average Diameter, D [nm]	First Standard Deviation, FSD [nm]	Variation Coefficient, VC [%]
CS-NP	15.4	4.1	26.4
CS/IgG-NP	79.3	20.2	25.7

**Table 2:** S(2p3/2), C(1s), N(1s), and O(1s) chemical environment of fabricated samples.

Sample	Spectra	Binding Energy (eV)	Bond	% Area
IgG protein	S(2p3/2)	164.1	C-S-S-C	69.5
		168.9	S=O	30.5
	C(1s)	284.9	CH-CH	22.1
		286.7	C-N, C=O	28.2
		288.2	N-C=O, O-C=O	49.7
	N(1s)	400	N-H	100
	O(1s)	531.9	O=C	39.8
		532.7	O-C	60.2
CS-NP	S(2p3/2)	--	--	--
	C(1s)	285.1	CH-CH, C-NH	15.5
		286.7	C-OH, C-O-C	47.7
		288.3	N-C=O, O-C-O	36.8
	N(1s)	399.6	N-H	5.8
		400.7	O=C-N	94.2
	O(1s)	531.5	O=C	39.8
		532.9	O-C	60.2
CS/IgG-NP	S(2p3/2)	--	--	--
	C(1s)	284.3	CH-CH, C-NH	3.7
		286.7	C-OH, C-O-C	21.9
		288.1	N-C=O, O-C-O	74.4
	N(1s)	398.8	C=N	1.8
		399.7	N-H	94.9
		401.1	NH_3_^+^	3.3
	O(1s)	531.4	O=C	76.8
		533.1	O-C	23.2

## Data Availability

Data are available by writing an e-mail to the corresponding author.
